# Nutritional Properties of Baobab Pulp from Different Angolan Origins

**DOI:** 10.3390/plants11172272

**Published:** 2022-08-31

**Authors:** Sara Monteiro, Fernando H. Reboredo, Maria Manuela Lageiro, Vanda M. Lourenço, João Dias, Fernando Lidon, Marta Abreu, António P. L. Martins, Nuno Alvarenga

**Affiliations:** 1 Faculdade de Ciências e Tecnologia, Campus da Caparica, Universidade Nova de Lisboa, 2829-516 Caparica, Portugal; 2 GeoBioTec Research Center, Faculdade de Ciências e Tecnologia, Campus da Caparica, Universidade Nova de Lisboa, 2829-516 Caparica, Portugal; 3 UTI, Instituto Nacional de Investigação Agrária e Veterinária IP, Quinta do Marquês, 2780-157 Oeiras, Portugal; 4 NOVA Math Research Center, Faculdade de Ciências e Tecnologia, Universidade Nova de Lisboa, 2829-516 Caparica, Portugal; 5 Instituto Politécnico de Beja, Rua Pedro Soares, 7800-295 Beja, Portugal; 6 LEAF Research Center, ISA, Universidade de Lisboa, Tapada da Ajuda, 1349-017 Lisboa, Portugal

**Keywords:** African food, *Adansonia digitata* L., baobab pulp, nutritional composition, elemental minerals, antioxidant activity, phenolic content, vitamin C

## Abstract

The baobab tree (*Adansonia digitata* L.) is found widely in the forests and savannas of sub-Saharan Africa. The baobab fruit has a sour and slightly sweet taste and is widely consumed by the natives, thus containing a high nutritional value and providing a source of income for rural people. This study aimed to compare the nutritional composition of baobab fruit pulp from different localities in the Namibe province (Angola). Twenty samples of baobab pulp were collected in markets of the four municipalities of Namibe. The results obtained showed that there is some geographic location dependence on nutritional and functional composition. The municipality of Camucuio showed samples with higher fibre content (56.62 g/100 g) and vitamin C (288.9 mg/100 g). Samples from the Virei municipality stood out for their antioxidant activity (1936 mmol TE/100 g), high K content (42.4 mg/g) and higher values of protein (2.42 g/100 g). The samples collected in the municipality of Bibala stood out for their high contents of carbohydrates (28.1 g/100 g), total phenolic compounds (972 mg GAE/100 g) and Ca (3.80 mg/g). Despite the differences in origin, the high nutritional value of baobab fruit has the potential to improve the diet of thousands of people in Africa qualitatively.

## 1. Introduction

The growing incidence of chronic diseases in industrial countries including cancer, diabetes and cardiovascular diseases has raised awareness regarding healthier habits, including regular exercise and a balanced diet [1]. Several vegetable food products have been identified as fundamental sources of bioactive compounds [2], presenting a significant impact on the prevention of such diseases, leading to a growing interest in the study of the type, concentration and effect of the different components on health [3].

African baobab (*Adansonia digitata* L.) belongs to the Malvaceae family and occurs in sub-Saharan Africa, including Tanzania, Sudan, Burkina Faso and Angola [4,5]. It is known as the “dead-rat tree”, “monkey-bread tree” [6] and also “arbre a palabre”, meaning the place in local communities where the elders get together to discuss problems [7].

The traditional use of baobab products (e.g., bark, fruits, seeds and leaves) contributes to the livelihood of local communities in Africa as a source of food, fibre and medicine [7], representing an important income generation [5]. In fact, the different parts of the plant have been used to treat several diseases, including diarrhoea, dysentery, malaria, fever and toothache [7,8]. Traditionally, baobab pulp is dissolved in water or milk to be used as a juice, sauce, snack or fermenting agent in local brews [5,6,9]. The sweet taste of pulp is due to the presence of fructose, saccharose and glucose, while the acidic character is due to organic acids such as citric, tartaric, malic, succinic and ascorbic [8]. The scientific community’s interest in baobab increased after the authorization of the European Union (EU) to place baobab dried fruit pulp on the market as a novel food ingredient in 2008 [10]. Later, in 2009, the U.S Food and Drug Administration (FDA) also recognized baobab fruit pulp as Generally Recognized as Safe (GRAS) [11].

Recent studies about the nutritional evaluation of different baobab parts (leaves, pulp and seeds) have revealed a high concentration of phenolic compounds [12,13], high antioxidant capacity [14] and essential fatty acids, vitamins (A, D, E and F) [12], at levels that fulfil the requirements of the daily recommended intake. Additionally, baobab pulp is characterized by low water content, high values of ascorbic acid, procyanidins, flavonol glycosides, dietary fibre, calcium (Ca) and potassium (K) [5,15,16,17,18,19]. The structure of polysaccharides extracted from baobab pulp is predominantly linear xylogalacturonans, whereas polysaccharides from leaves are mostly pectic polysaccharides with a 1.5:1 ratio of homogalacturonan to rhamnogalacturonan-I [20]. The compact conformation of polysaccharides from baobab pulp at the emulsions’ interface allows for more considerable stability unveiling a potential new source of stabilizers for the food industry [21]. Consequently, producing new ingredients extracted from baobab with a positive impact on health may present a new opportunity for developing food products with added value [21].

However, significant variations have been observed in the reported value of baobab parts [22,23] which may be due to different mixtures on samples obtained from the market, the age of the population, the analytical methods and storage conditions, among others [22]. Although the information is still scarce, earlier studies indicate an influence of the provenience of baobab trees on the nutrient content of pulp [5,24], in part as a consequence of physicochemical soil characteristics [24].

Most recent studies on baobab fruit rely on populations located in south and eastern Africa, such as Kenya, Sudan [4,25] and South Africa [26], although some studies have been performed in western Africa such as Mali [15] and Nigeria [12]. Despite the baobab’s importance to the local economy, information concerning the nutritional characterization of baobab pulp is still scarce in Angola. Therefore, this study aimed to evaluate the impact of the geographical provenance of baobab fruits, from Namibe province (southern Angola), on the mineral content and antioxidant potential of baobab pulp.

## 2. Results

### 2.1. Nutritional Characterization

Table 1 shows the mean values and standard deviations for each constituent and municipality, the Figure S1 of the supplementary materials shows the global distribution of the different baobab constituents across the four Namibe municipalities. There are some constituents and municipalities where the order of magnitude of the mean and standard deviation is the same. Looking at the individual samples data (not shown), specifically vitamin C, total phenolic content (TPC), antioxidant activity (AA) and calcium (Ca) from the Virei (VSI) municipality in the Namibe southern inland, we observe that the TPC and AA levels from Cahinde community are much higher (one order of magnitude greater than the similar data from the Virei community (both communities make part of the Virei municipality). In the case of vitamin C and TPC from the Camucuio (CNI) municipality in the Namibe northern inland, we detect one outlying observation in the Comucuio and Mamué locations, respectively. For TPC and AA from the Bibala (BCI) municipality in the Namibe centre inland, the problem arises from one of Bibala’s locations, where the recorded observations are one order of magnitude greater than the remaining. Finally, one community of the Moçâmedes (MC) municipality in the Namibe coastline has lower levels of both antioxidant activity (AA) and phosphorus (P) when compared with the results from the remaining four municipalities.

### 2.2. Principal Component Analysis (PCA)

Principal component analysis (PCA) was carried out (Table 2) in order to find eventual regional (or ecotype) effects on the nutritional characteristics of the baobab pulp. The main nutritional attributes (moisture, ash, TDF, TPC, AA and vitamin C, and the minerals calcium (Ca), potassium (K) and phosphorus (P)) were the considered variables for PCA and the regression process.

The similarity map defined by the first two principal components accounted for 71.4% of the total variance. The first component (PC1) condensed 52.47%, and the second component (PC2) represented 18.90% of the total variance.

**Figure 1:**
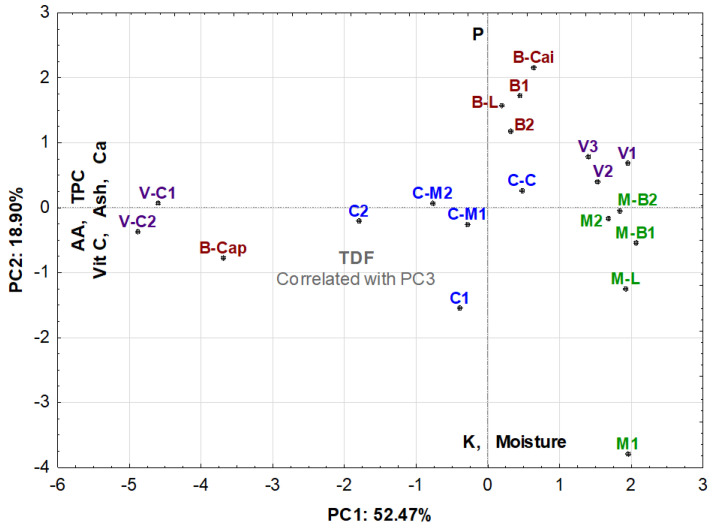
Principal component analysis projection of the samples.

The PC1 was heavily loaded with ash, Ca, vitamin C, TPC and AA attributes, all negatively loaded. The PC2 correlates negatively with moisture and K and positively with P. The total dietary fibre (TDF) was inversely correlated with PC3. Figure 1 shows that the projection of the samples onto the PC1/PC2 plane. The Ca vector had no significant correlation with any of the components.

The positive correlations between the mineral (for Ca) and bioactive composition (vitamin C, TPC, AA) of the fruit suggest that possible differences in soil-available mineral nutrition in the tested regions may impact the quality of the fruit.

The PCA analysis allows us to highlight the following findings:

1—It was possible to cluster samples from small villages and also from different municipalities;

2—It allows us to confirm an ecotypic, almost geographical similarity between samples, namely samples V1, V2 and V3; V-C1 and VC2, B1 and B2; C-M1 and C-M2; M-B1 and M-B2;

3—In terms of minerals, samples from Bibala (except the sample picked in Capangombe) presented a high amount of P and a low amount of K;

4—Samples from Cainde (a small village of Virei) and Capangombe (a small village of Bibala) presented a high content of Ca and a high bioactive composition (AA, TCP and vit C);

5—Generally, all the samples picked in Moçamedes, in the Town of Virei and Bibala (except the sample picked in Capangombe) had poorer bioactive contents.

## 3. Discussion

The results show significant differences among the samples obtained in the different municipalities regarding the levels of protein, moisture, ash, sugars, vitamin C, total phenolic compounds (TPC), antioxidant activity (AA) and elemental minerals. In the case of TDF, carbohydrates and energy, no significant differences were noted.

Regarding moisture content, baobab fruit pulp presented values ranging between 12.5% in BCI and 13.8% in MC. According to the criteria established by the European Union Legislation [10], the moisture contents of baobab fruit pulp ranged from 11.1% to 12.0%, values a little bit lower when compared to those found in the current study. This moisture content is a little high for the powdered product and can negatively influence its shelf life [22]. The variation in moisture content can be attributed to sampling origin, quality and age, plus storage conditions. The moisture content of baobab pulp reported in the literature varies widely among authors, from 2% to 27% [22].

Our data on moisture content are with some exceptions similar to those published previously [22,23,27,28,29,30]. Conversely, our results were higher when compared to the values reported by [5,31,32,33,34]. The low moisture content in baobab pulp mentioned in the literature can be attributed to low altitude, relatively high temperature and low precipitation, beyond other factors such as sun and wind exposure that may well contribute to the dehydration of the fruit pulp [35].

The ash levels varied slightly among the samples from different municipalities. The ash content of baobab pulp was higher in samples from VSI and CNI (5.18% and 5.01%, respectively) and lower in samples from MC and BCI (4.71% and 4.75%, respectively). Since the ash content reflects the inorganic matter, baobab pulps from VSI and CNI municipalities are richer in minerals than baobab from BCI and MC municipalities.

The ash content of baobab pulp established by the European Union [10] ranges between 5.5–6.6%. Our results are in agreement with others described in the literature [22,27,28,31,32], but contrast with the average ash content of 1.9% reported by [36]. This difference may be due to the different methods used by the authors, the variation in the samples’ incineration time and the variation in the temperature used [22].

The protein content of baobab fruit pulp from different municipalities (BCI, CNI, MC and VSI) ranged from 2.10% to 2.42% (m/m), thus within the limits of 2.03–3.24% of the European Union evaluation [10] regarding a novel food ingredient. Previous studies on baobab fruit pulp showed somewhat higher protein contents, between 2.5% and 3.6%, but of the same order of magnitude [24,27,28,31,32]. It should be noted that baobab is a fruit characterized by low protein and fat content, and consumption habits add water, thus diluting the existing protein levels.

At the municipality level, the mean total dietary fibre (TDF) content ranged from 51.38% in the fruit pulp of MC municipality to 56.62% in the pulp of CNI municipality. Furthermore, no significant differences in the TDF content were observed within the studied areas, showing that the effect of environmental conditions on the TDF content is negligible. In previous work, Muthai et al. [5] stated that the crude fibre and cellulose content is not influenced by the samples’ provenance (West, East or South Africa). It may be likely that this is also the case for dietary fibre.

The fibre content of baobab fruit pulp mentioned in the literature [5,24,27,28,32] is generally <11.15% with levels ranging from 5.4% to 11.15%. However, others point out values ranging from 45.1% to 52% [31,37,38]. These values of fibre contents are similar to our data. This discrepancy is related with different evaluations, crude fibre or cellulose instead of dietary fibres, which is not the same. However, the use of different analytical methods also influenced the final results because the studies that obtained high fibre values used the enzymatic gravimetric method, while the others used the AOAC method [39] or the acid–alkaline hydrolysis method [22]. The main difference between these methods is that the enzymatic gravimetric method captures much more fibre fractions, including lignin, cellulose, hemicellulose, pectin and resistant starch [23].

Regarding the concentration of carbohydrates in the baobab fruit pulp ranges from 22.91% (CNI municipality) to 28.06% (BCI municipality), our data is far from the levels recorded in both the literature, at between 37.00% and 88.60% [23,24,27,28,31,33,37] and the European Union evaluation [10] regarding a novel food ingredient which indicates levels between 78.3% and 78.9%.

These differences in carbohydrate concentrations are due to the fact that fibre content increases the apparent amount of digestible carbohydrate because carbohydrate is calculated by the mass remaining after protein, fat, ash and fibre are subtracted from 100 g of sample [31]. As in this study, dietary fibre was determined, and the values ranged from 51.38% to 56.62%. The carbohydrate values were mandatorily lower, ranging from 22.91% to 28.06%. Most authors determined the carbohydrates by difference, so these values are not expected to be very accurate [22]. It is clear that baobab pulp is an excellent source of carbohydrates despite the low concentrations reported in the current study.

The energy values ranged from 1208 kJ/100 g to 1262 kJ/100 g. In general, the energy value of all baobab pulp samples was high when compared to the energy values published by [31,34], who mentioned energy values in the range of 846 kJ/100 g to 849 kJ/100 g, although close to other values reported by [23,28,30], who recorded energy values in the range of 1380 kJ/100 g to 1494.9 kJ/100 g.

These differences in energy levels are highly dependent on the carbohydrate content [30]. In addition, the coefficients used by authors to calculate the energy value are sometimes slightly different [22].

Regarding the calcium (Ca) concentrations in baobab pulp samples, the values ranged from 2938 mg/kg to 3797 mg/kg. The high Ca content makes the fruit attractive as a natural source of Ca supplementation for pregnant and lactating women, as well as for children and the elderly, because Ca is an essential mineral for bone structure and function [28,40]. The obtained Ca levels were higher than those reported by other authors in baobab pulp [5,24,27,30,40], who recorded values in the range of 600 mg/kg to 2500 mg/kg, although [32] had noticed a value of 6550 mg/kg.

The potassium (K) concentrations in baobab pulp did not vary significantly among the different municipalities; the highest K content was recorded in the sample from VSI municipality (42.37 mg/g). The lowest level was observed in the sample from the BCI municipality (37.53 mg/g). The baobab fruit pulp is an excellent source of K, an essential mineral for the functioning of the body, as it helps regulating muscle contraction and nerve impulse transmission [5]. The results obtained in this study are higher when compared to those published by other authors who mention levels in the range of 7.26 mg/g to 28.36 mg/g [5,24,30,40].

In the case of phosphorus (P), the samples from the different municipalities exhibited high P concentrations with values ranging from 9279 mg/kg (MC) to 11,355 mg/kg (VSI). The results indicate that baobab pulp is a good source of P, an element essential for human growth and healthy maintenance of bones, teeth and muscles [5].

The P values reported in the literature are generally between 450 mg/kg and 4520 mg /kg [27,30,32,40,41], far behind the data of the current study. In general, the mineral content of baobab pulp reported in the literature shows significant variability. Some authors claim that these differences can be attributed to the composition of the soils, climatic factors, and the use of different methods [22,42].

The concentrations of sulphur (S) ranged between 1674 mg/kg (MC) and 2163 mg/kg (VSI), although there is a consistent lack in the literature regarding studies evaluating the levels of this element in baobab pulp which is also extensive to chlorine (Cl). Sulphur is essential for forming connective tissue, forming sulfur amino acids and antioxidants such as glutathione [43]. Chlorine is key in regulating body fluids, electrolyte balance, the preservation of electrical neutrality and acid–base status, thus the second most abundant electrolyte in serum [44].

Regarding the total phenolic content (TPC) of baobab fruit pulp, a significant variability among the samples was observed–the pulp from BCI municipality had the highest content (972.2 mg GAE/100 g) while the sample from MC municipality had the lowest content (460.5 mg GAE/100 g). The observed levels are similar with the ones reported by [14,45,46] that presented TPC between 1090 to 379.48 mg GAE/100 g; however, these values are smaller than the ones presented by Tembo et al. and Lamien-Meda et al. [47,48], who reported values ranging from 1870 mg GAE/100 g to 4057.5 mg GAE/100 g.

These variations can be attributed to climatic factors and also the extraction solvent that was used. A study on baobab fruit pulp with different solvents revealed that TPC was significantly higher in acetone extract than in methanol extract. Previous studies have stated that the nature of the solvent influences the extraction efficiency and the content of phenolic compounds [47,48]. In addition, some procedures applied by some authors, such as freezing the samples at −20 °C before analysis, may have influenced the TPC results. Asami et al. [49] concluded that freezing fruit samples may lead to higher extraction efficiency of total phenolic compounds due to greater rupture of plant cells during ice formation.

Regarding the vitamin C content, the samples of baobab fruit pulp from the different municipalities (BCI, CNI, MC and VSI) showed significant variations. The sample from MC municipality had the lowest level (163.8 mg/100 g), while the sample from CNI municipality had the highest content (288.9 mg/100 g). This difference in vitamin C content can be attributed to climatic factors, soil type and sunlight intensity between the study areas [50]. Several studies have documented varying levels of vitamin C content in baobab fruit pulp, with mean values ranging from 126 mg/100 g to 509 mg/100 g [22,32,34,38,47,51,52]. The results obtained in this study are therefore within the range reported by those authors.

Another possible explanation for a high vitamin C content variation is related to storage condition and duration, as vitamin C is labile to light and heat [23]. Traditionally, the baobab fruit pulp is mixed in water or milk, and the liquid obtained is used as a refreshing drink or as a sauce in food preparation. In addition, the baobab fruit pulp is also used as a fermentation substrate in local brewing or as a substitute for cream of tartar in baking [9], cited by De Caluwé et al. [17]. Recently in urban areas, the pulp of the baobab fruit has become a popular ingredient and has been used in the preparation of jam, ice cream, jelly, biscuits, breakfast and cereals. However, to retain the vitamin C activity, the baobab fruit pulp must be added to foodstuffs after removal from the heat source.

Chadare et al. [22] stated that consuming 40 g of baobab fruit pulp provides 100% of the recommended daily intake of vitamin C for pregnant women (19–30 years). It is important to note that generally such calculations are made considering the digestibility and bioavailability of nutrients.

## 4. Materials and Methods

### 4.1. Samples Collecting and Treatment

Namibe is one of the 18 provinces of Angola, located in the country’s southern region. It has a land area of approximately 57,091 km^2^ and an Atlantic maritime border of 480 km. It is bounded to the north by the province of Benguela, east by the province of Huila, west by the Atlantic Ocean and south by the Cunene River and the Republic of Namibia. The Namibe province consists of five municipalities: Moçâmedes (MC), Virei (VSI), Bibala (BCI), Camucuio (CNI) and Tômbwa, the last one without the presence of baobab trees.

**Figure 2:**
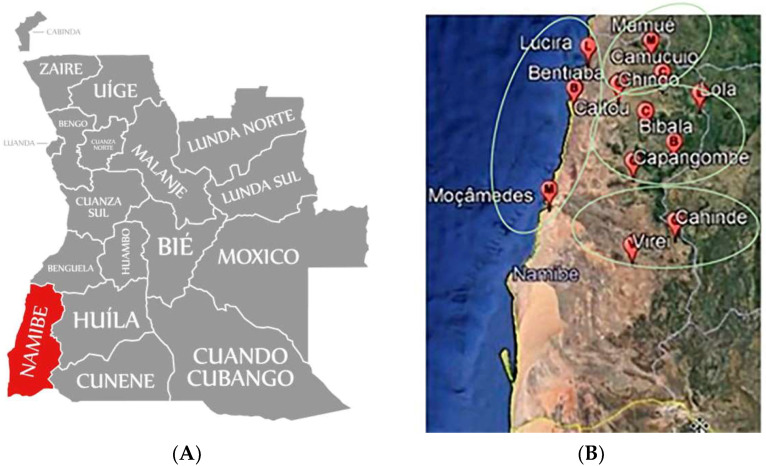
(**A**) Map of the Namibe province in Angola [53] and (**B**) map of sampling locations in four Namibe communities [54].

The samples from different Angola locations in the Namibe province, Figure 2, located in 4 different municipalities and collected in 5 different community markets for each municipality, were kept separately and packed in polyethylene sampling bags, kept inside isothermal boxes and transported by air to Lisbon (Portugal) for further processing and analysis. The GPS locations of the samples collected are shown in Table 3. Subsequently, the baobab packages were transported to the National Institute for Agricultural and Veterinary Research, IP (INIAV) laboratory. Baobab flour was prepared from the baobab pulp according to the following methodology: the baobab pulp was grounded in a food processor (Thermomix Tm31, Vorwerk, Germany), and then a 120 µm mesh sieve was used to separate the flour from the fibres and seeds and then, in order to obtain a more refined flour, 150 µm and 170 µm mesh sieves were used.

### 4.2. Analitical Methods

#### 4.2.1. Baobab Macro Nutrients Characterization

Moisture expressed as a sample percentage (%, *w/w*) was determined in duplicate using a gravimetric method by drying 2.0 g of each sample in an oven (Memmert, U50, Schwabach, Germany) at 103 °C to constant weight, as mentioned by Assogbadjo et al. [24].

The ash content (%) was determined in duplicate by the gravimetric method after incineration of 2 g of sample in a muffle furnace (Thermolyne Muffle Furnace, model 48000, Dubuque, IA, USA) at 550 °C for 8 h according to Godočiková et al. [55].

Total nitrogen was determined according to the Kjeldahl method as described by [56] with some modifications. Briefly, 1.0 g of each sample was digested in sulfuric acid with a catalyst (potassium sulphate and copper sulphate) with a temperature increase to 430 °C for 2 h. The acid digestion took place in a digestor (Foss, Tecator 2020 Digestor, Hillerød, Denmark). Then distillation was performed in the automatic distiller (Foss, 2300 Kjeltec analyzer unit, Hillerød, Denmark) with sodium hydroxide (40%) and the distilled ammonia, using bromocresol green and methyl red as indicators in reaction with boric acid (2%). Afterwards, the titration with hydrochloric acid (0.1 N) was carried out. Once the total nitrogen in the samples was determined, the crude protein content was calculated using a conversion factor of 6.25 to convert the nitrogen percentage to the crude protein percentage. Each sample was processed in duplicate, and the results were expressed as a sample percentage (%).

The total dietary fibre (TDF) content was determined in duplicate by an enzymatic-gravimetric procedure based on AACC method 32-05.01 and AOAC method 985.29 and according to Prosky et al. [39] using the Megazyme fibre kit K-TDFR. Briefly, the samples were dried in an oven at 105 °C overnight and cooled in the desiccator. To a 400 mL precipitation beaker, about 1.0 g of dried sample was weighed in duplicate. Then 50 mL of phosphate buffer (pH 6.0) was added. Subsequently, the samples were gelatinized by incubation with α-amylase thermostable and then enzymatically digested with protease and amyloglucosidase to remove protein and starch which was followed by two incubations in a thermostated bath (J P Selecta, Unitronic 320, Barcelona, Spain). Then the total dietary fibre was precipitated with 280 mL 96% ethanol, and a filtration system filtered the residue with vacuum pump (Gast, model D0A-P104-BN, Benton Harbor, MI, USA). Finally, the residue was washed with 78% ethanol, 95% ethanol and acetone. The dried residue was weighed. One sample duplicate was analysed for protein determination (Kjeldahl method), and another was incinerated at 525 °C for 5 h for ash content determination. The total dietary fibre corresponds to the mass of the residue in percentage after digestion minus the value for protein and ash, also rectified by subtracting the value of the blank test.

The fat was obtained by hot solvent extraction with petroleum ether for about 4 h using a Soxhlet extractor according to the standard NP ISO 6492, 2014 [57]. After extraction, traces of the solvent used were allowed to evaporate at room temperature and then the fat was kept in an oven (Memmert, model U50, Schwabach, Germany) at 103 °C for 1 h. Subsequently, after cooling in, the desiccator was weighed. The total fat yield was determined in duplicate and expressed as a percentage, as mentioned by [58] in a previous assay using baobab samples.

The total carbohydrate content was estimated by difference using the following formula: carbohydrates (%) = 100 − [moisture (%) + protein (%) + fat (%) + ash (%) + fibre (%)], the medium value of 8.9 ± 0.05% was used for fat.

The baobab pulp energy evaluation, in kcal/100 g, was calculated using conversion factors based on general Atwater factors for food energy content in EU Regulation N. 1169, 2011 [59], being 4.0 kcal/g for protein and carbohydrates; 9.0 kcal/g for fat and 2.0 kcal/g for fibre [28].

#### 4.2.2. Elemental Analysis

The determination of micro and macronutrients in pulp baobab samples, expressed in mg/kg, was performed in triplicate by using an X-ray analyzer (Thermo Scientific, Niton model XL3t 950 He GOLDD+, Waltham, MA, USA) has described in Fernandes et al. and Reboredo et al. [60,61]. Detection limits using the optimum mining mode for a period of 120s under high purity helium atmosphere were: Ca = 65 mg/kg; Cl = 75 mg/kg; K = 200 mg/kg; P = 450 mg/kg; S = 90 mg/kg; Plant reference materials were used for data validation: orchard leaves (SRM 1571) and poplar leaves (GBW 07604) were run before the beginning of analyses and after every five samples. The recovery values ranged between 90 and 97%.

#### 4.2.3. Total Phenolic Content (TPC) and Antioxidant Activity (AA) Determination

Baobab extracts for total phenolic content (TPC) and antioxidant activity (AA) were prepared in duplicate according to the protocol described by Cerit et al. [62] with some modifications. About 0.5 g of each sample was extracted with 20 mL methanol (100%). Then the samples were homogenized in a polytron (Ika, Ultra-Turrax T25, Staufen, Germany), placed for 5 min in an ultrasonic bath (Bransonic, Branson 5200, Branson, MO, USA) and incubated overnight at 4 °C. Subsequently, the mixtures were centrifugated at 4500 g for 20 min at 4 °C in a centrifuge (Sigma, 2K15, Osterode am Harz, Germany). The supernatants were filtered with a Whatman 41 filter paper and stored at −20 °C for further analysis. Total phenolic content (TPC) was determined spectrophotometrically by the Folin–Ciocalteu method with some modifications [63,64,65,66]. A volume of 50 μL of the sample extract was mixed with 150 μL of diluted Folin-Ciocalteu reagent. Next, 100 μL of methanol 100% was added to the mixture, and then 2400 μL of distilled water was added. After 3 min, 300 μL of diluted sodium carbonate (Na_2_CO_3_, 1 N) was added to the mixture and incubated for 2 h in the dark at room temperature. The absorbance was measured at 725 nm using a spectrophotometer (Thermo Fisher Scientific, Unicam UV4, Waltham, MA, USA). Gallic acid was used as a standard, and the results were expressed as mg of gallic acid equivalent (GAE) per 100 g of sample.

The antioxidant activity (AA) was determined using the DPPH (2,2-diphenyl-1-picrylhydrazyl) method according to the procedures described by Brand-Williams et al. [67] with some modifications. A volume of 50 μL of sample extract was added to 2850 μL of diluted DPPH. This was followed by adding 100 μL of methanol. After 2 h in the dark at room temperature, the absorbance was measured at 580 nm using a spectrophotometer (Thermo Fisher Scientific, Unicam UV4, Waltham, MA, USA). A calibration curve was prepared using trolox as standard in the range of 25 to 800 µM. The results were expressed as mmol of trolox equivalent (TE) per 100 g of sample.

#### 4.2.4. HPLC Quantification of Vitamin C

High-pressure liquid chromatography (HPLC) determined the vitamin C content as described in the European Standard EN 14130, 2003 [68]. To extract the baobab vitamin C, 1.5 g of each sample was weighed, and 40 mL of metaphosphoric acid solution (2%) was added, sample extraction was performed in duplicate. It was then stirred for 15 min and filtered through a rapid paper filter under vacuum. The dehydroascorbic acid was converted to ascorbic acid by addition of aqueous cysteine solution (4%), trisodium phosphate solution (20%) (till reach pH between 7.0 to 7.2), stirring for 5 min and addition of metaphosphoric acid solution (20%) to decrease pH between 2.8 to 2.5. Finally, the supernatant was collected and filtered through a 0.45 µm nylon filter, and 50 µL of extract from each sample was injected. Each extraction was performed in duplicate. The analyses were performed on an Alliance 2695 HPLC system (Waters, Milford, MA, USA) with a Waters 2996 photodiode array detector (PDA), using a Waters Spherisorb ODS2 column (4.6 × 250 mm, 5 μm). The mobile phase consisted of 13.6 g potassium dihydrogen phosphate dissolved in 900 mL water mixed with 1.82 g N-cetyl-N, N, N-trimethylammonium bromide dissolved in 100 mL methanol. The column was kept at 25 °C, and ascorbic acid was detected at 265 nm. The total analysis time was 10 min at a flow rate of 0.8 mL min^−1^. The peak areas were quantified and processed with version 2.0 of the Empower Software (Waters, Milford, MA, USA) by comparison to a calibration from 0.5 to 30.0 ppm ascorbic acid standard, with linear regression [area (UA) = 155,108 (UA/ppm) × vitamin C (ppm)–70,392 (UA)], with R² = 0.9995, being 23.5 mg/100 g for the limit of detection and 71.3 mg/100 g of sample for the limit of quantification.

### 4.3. Statistical Methods–Multiple Comparisons

The statistical analysis of the data referring to the multiple comparisons of the constituent’s means across municipalities was performed using the statistical software R, version 4.1.2 (GNU General Public License, Boston, MA, USA). The hypothesis tests for assessing the pairwise differences in the constituent means across the municipalities are as follows:

(1) $$H_{0}:\mu_{i}=\mu_{j}{\;\mathrm{vs.}\;H}_{1}:\mu_{i}\neq \mu_{j}\;\forall \;i\neq j.\;$$

with i, j ∈ {BCI, CNI, MC, VSI}, N = n _BCI_ = n _CNI_ = n _MC_ = n _VSI_ = 5, and μ refers to the population mean of the constituent’s concentration. This test is preceded by the test which assesses that there are at least two significant differences of means amongst the four municipalities, i.e.,

(2) $$H_{0\;}:{\;\mu }_{i}=\mu_{j}{\;\mathrm{vs.}\;H}_{1\;}:\;\exists \;i\neq j\;:{\;\mu }_{i}\neq \mu_{j}.$$

The choice of the adequate statistical testing procedures for testing these hypotheses depends on the validation or not of the normality assumption of the constituent’s concentrations within municipality as well as on the homogeneity of variances of the constituent’s concentrations across municipalities. In this way, the normality assumption was checked for all the constituents within each municipality via the Shapiro–Wilks normality test (Table S1 of supplementary materials) [69]. The homogeneity of variances across municipalities assumption was checked by the Bartlett test, in the case of data normality within all municipalities [70], and Levene’s test test, in the case of non-normality of observations within all municipalities [71] (Table S2 of supplementary materials).

For mean comparison (Hypotheses (2) and (1) in this order of baobab’s constituents across the four Namibe municipalities, was used the F-ANOVA plus the Tukey tests, in the case of data normality within the municipality and variance homogeneity across municipalities [72], the F-Welch–ANOVA plus the Dunnet–Tukey–Kramer tests, in the case of data normality within municipality but no variance homogeneity across municipalities [73,74], and the non-parametric Kruskal–Wallis plus Dunn tests, in the case of no data normality within municipality but variance homogeneity across municipalities; [75,76] (Table S3 of supplementary materials). We assumed all samples to be independent and considered the 5% significance level for deciding against the null hypothesis in all tests.

### 4.4. Principal Component Analysis

Principal component analysis (PCA) was performed in order to search possible geographical influences on nutritional properties of baobab using STATISTICA 8.0 (StatSoft, Tulsa, OK, USA).

## 5. Conclusions

Due to its nutritional and functional composition, baobab pulp seems suitable for large-scale regional and global marketing as food or as an ingredient to improve food functionality, mostly based on the high vitamin C content, dietary fibre, antioxidant activity and phenolic content, plus a rich source of calcium and potassium. However, the levels of the nutrients can be influenced by the location of the collected baobab samples, an issue that should be addressed in further investigation. The low moisture content of this fruit promotes its good preservation capacity, which in terms of post-harvest availability, is an excellent opportunity to supply the food shortages of the local population.

This study provides information for health professionals (nutritionists) and food policymakers in Africa to encourage the population to consume baobab pulp and promote the preservation of the baobab tree. Finally, our data is intended to fill a gap in the literature regarding the lack of information about the nutritional composition of baobab pulp of Angolan origin.

## Figures and Tables

**Table 1:** Means and standard deviation (within brackets) of the different baobab’s constituents across the four Namibe municipalities and collected samples, and results from the pairwise tests on the equality of means of the constituents across the four municipalities (Equation (2)—Tukey, Dunnet-Tukey-Kramer and Dunn tests) for the constituents for which the existence of at least one pairwise significant difference was detected (Equation (1)—F-ANOVA, F-Welch-ANOVA and Kruskal–Wallis tests).

Component/Municipality	BCI	CNI	MC	VSI
**Moisture (%)**	12.50 (0.76) ^b^	13.20 (0.32) ^ab^	13.81 (0.37) ^a^	12.91 (0.36) ^ab^
**Ash (%)**	4.75 (0.23)	5.01 (0.27)	4.71 (0.09)	5.18 (0.63)
**Protein (%)**	2.24 (0.04)	2.10 (0.18)	2.27 (0.03)	2.42 (0.20)
**TDF (%)**	52.17 (2.86) ^a^	56.62 (1.84) ^a^	51.38 (1.80) ^a^	54.21 (4.88) ^a^
**HC (%)**	28.1 (2.5)	22.9 (1.7)	27.6 (1.8)	25.1 (5.3)
**Energy (kJ/100 g)**	1 262 (21) ^a^	1 208 (15) ^b^	1 249 (16) ^ab^	1 230 (48) ^ab^
**Ca (ppm)**	3 797 (862)	3 696 (425)	2 937 (352)	3 409 (1 019)
**K (ppm)**	37 528 (3 642)	41 834 (3 780)	40 973 (3 400)	42 368 (4 520)
**P (ppm)**	10 805 (768)	10 010 (766)	9 279 (1 791)	11 355 (140)
**Cl (ppm)**	36 081 (2 858)	33 999 (2 376)	31 997 (5 284)	38 273 (629)
**S (ppm)**	1 960 (176) ^a b^	1 864 (165) ^ab^	1 675 (324) ^b^	2 164 (180) ^a^
**TPC** **(mg GAE/100 g)**	972.2 (427.2) ^a^	758.4 (168.6) ^ab^	460.5 (88.7) ^b^	872.8 (602.8) ^ab^
**AA** **(mmol TE/100 g)**	1 834 (1 360) ^a^	1 406 (338) ^a^	536.9 (161.3) ^b^	1 936 (1 599) ^ab^
**Vitamin C (mg/100 g)**	284.6 (38.4) ^a^	288.9 (130.2) ^ab^	163.8 (12.8) ^b^	251.6 (116.7) ^ab^

Significant differences are marked with different letters in each line. The same letter means no differences in means were detected. Detailed results from these tests and the preceeding normality and variance homegeity tests can be found in the Supplementary Materials.

**Table 2:** Principal component analysis.

Component	PC1	PC2	PC3
Vitamin C	−0.82 **	0.11	−0.12
TPC	−0.92 **	0.00	0.22
Ca	−0.84 **	−0.14	0.19
K	−0.59	−0.71 **	−0.08
P	−0.40	0.68 *	0.31
Moisture	0.30	−0.82 **	0.20
Ash	−0.89 **	−0.15	−0.18
TDF	−0.45	0.12	−0.79 **
AA	−0.95 **	0.00	0.25
Eigenvalue	4.72	1.70	0.96
% Variance	52.47	18.90	10.64
% Cumulative variance	52.47	71.37	82.01

* Marked values were considered moderately correlated with the PC; ** marked values were considered strongly correlated with the PC, following the classification used previously [27,28,29].

**Table 3:** GPS location of the baobab samples collected in Namibe of Angola.

Municipality	Location in Namibe	Community	Sample Code	GPS Location
Bibala (BCI)	Center inland	Bibala	B1	14°45′42.8″ S 13°21′21.2″ E
			B2	
		Caitou	B-Cai	14°28′49.0″ S 13°04′52.9″ E
		Capangombe	B-Cap	14°56′31.4″ S 12°57′43.4″ E
		Lola	B-L	14°18′15.7″ S 13°36′16.8″ E
Camucuio (CNI)	Northern inland	Chingo	C-C	14°13′00″ S 12°49′00″ E
		Camucuio	C1 C2	14°06′47.8″ S 13°14′35.9″ E
		Mamué	C-M1 C-M2	13°49′00″ S 13°08′00″ E
Moçâmedes (MC)	Coastline	Bentiaba	M-B1 M-B2	14°15′33.1″ S 12°23′26.6″ E
		Lucira	M-L	13°52′05.4″ S 12°31′34.4″ E
		Moçâmedes	M1 M2	15°12′20.0″ S 12°08′51.7″ E
Virei (VSI)	Southern inland	Cahinde	V-C1 V-C2	15°29′51.8″ S 13°22′11.7″ E
		Virei	V1 V2 V3	15°43′33.4″ S 12°57′04.1″ E

## Data Availability

Data are contained within the article or supplementary material.

## Supplementary Materials

The following supporting information can be downloaded at: https://www.mdpi.com/article/10.3390/plants11172272/s1. Figure S1. Global distribution of the different baoba’s constituents across the four Namibe municiplaties. Table S1. P-values obtained from the Shapiro-Wilks normality tests for each of the constituents’ levels within each municipality. Grey cells indicate those tests that failed at the 5% significance level. Table S2. P-values obtained from the Bartlett and Levene tests of variance homogeneity for each of the constituents’ levels across the 4 municipalities. Grey cells indicate those tests that failed at the 5% significance level. Table S3. Results from the pairwise comparison of means across the 4 municipalities for the constituents for which the existence of at least one pairwise significant difference was detected by the F-ANOVA, F-Welch-ANOVA and Kruskal Wallis tests. Significant differences are marked with X.
