# Supplementary material for: Nutritional Properties of Baobab Pulp from Different Angolan Origins
Source: Plants (Basel). 2022 Aug 31;11(17):2272. doi: 10.3390/plants11172272 (PMC9460372; doi:10.3390/plants11172272)
Supplement: Supplementary file 1 [file plants-11-02272-s001.zip › plants-1858345-supplementary.pdf]

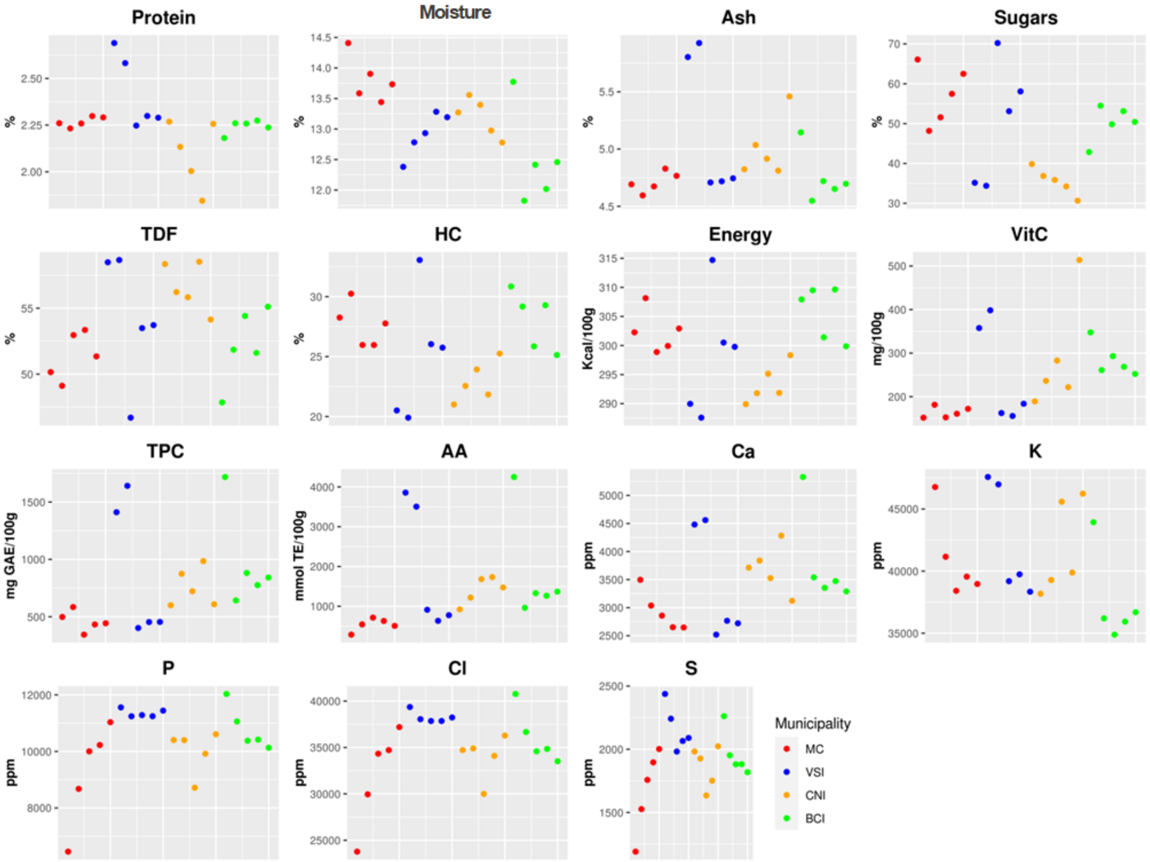

Figure S1. - Global distribution of the different baoba's constituents across the four Namibe municiplaties.

Table S1. P-values obtained from the Shapiro-Wilks normality tests for each of the constituents' levels within each municipality. Grey cells indicate those tests that failed at the 5% significance level.

Constituint/Municipality	BCI	CNI	MC	VSI
**Moisture (%)**	0.1847	0.8075	0.5690	0.7083
**Ash (%)**	0.1009	0.1020	0.9502	0.0196
**Protein(%)**	0.1860	0.4924	0.5881	0.1353
**TDF (%)**	0.5598	0.4989	0.6465	0.3080
**HC (%)**	0.3779	0.8669	0.4265	0.4161
**Energy (kcal/100g)**	0.1095	0.5403	0.4909	0.5258
**Ca (ppm)**	0.0035	0.9853	0.2840	0.0399
**K (ppm)**	0.0167	0.1332	0.0812	0.0642
**P (ppm)**	0.2484	0.0892	0.4663	0.1697
**Cl (ppm)**	0.2571	0.1829	0.4581	0.0324
**S (ppm)**	0.0635	0.45990	0.6782	0.5471
**TPC (mg GAE/100g)**	0.0312	0.4192	0.9409	0.0416
**AA (mmol TE/100g)**	0.0035	0.5793	0.7365	0.0416
**Vitamin C (mg/100g)**	0.2185	0.0587	0.4341	0.0758

Table S2. P-values obtained from the Bartlett and Levene tests of variance homogeneity for each of the constituents' levels across the 4 municipalities. Grey cells indicate those tests that failed at the 5% significance level.

	p-value
**Moisture (%)**	0.2608
**Ash (%)**	0.2915
**Protein (%)**	0.0009
**TDF (%)**	0.1665
**HC (%)**	0.0767
**Energy (kcal/100g)**	0.0665
**Ca (ppm)**	0.5799
**K (ppm)**	0.3012
**P (ppm)**	0.0016
**Cl (ppm)**	0.8841
**S (ppm)**	0.4745
**TPC (mg GAE/100g)**	0.3704
**AA (mmol TE/100g)**	0.3219
**Vitamin C (mg/100g)**	0.0013

Table S3. Results from the pairwise comparison of means across the 4 municipalities for the constituents for which the existence of at least one pairwise significant difference was detected by the F-ANOVA, F-Welch-ANOVA and Kruskal Wallis tests. Significant differences are marked with X.

	Paired municipalities					
	**VSI-BCI**	**CNI-BCI**	**CNI-VSI**	**MC-VSI**	**MC-CNI**	**MC-BCI**
Moisture (%)				X		X
Energy (kcal/100g)		X				
S (ppm)				X		
TPC (mg GAE/100g)						X
AA (mmol TE/100g)					X	X
Vitamin C (mg/100g)						X
